# The effect of Camellia sinensis ointment on perineal pain and episiotomy wound healing in primiparous women: A triple-blind randomized clinical trial

**DOI:** 10.1371/journal.pone.0305048

**Published:** 2024-08-01

**Authors:** Masoumeh Sayahi, Azam Jahangirimehr, Zahra Hatami Manesh, Faraz Mojab, Maryam Nikbina

**Affiliations:** 1 Department of Midwifery, Shoushtar Faculty of Medical Sciences, Shoushtar, Iran; 2 Department of Public Health, Shoushtar Faculty of Medical Sciences, Shoushtar, Iran; 3 School of Nursing and Midwifery, Dezful University of Medical Sciences, Dezful, Iran; 4 Department of Pharmacognosy, School of Pharmacy, Pharmaceutical Sciences Research Center, Shahid Beheshti University of Medical Sciences, Tehran, Iran; Kasr Alainy Medical School, Cairo University, EGYPT

## Abstract

**Background and objective:**

Episiotomy is one of the most commonly performed procedures in obstetrics. complications of episiotomy are pain, bleeding, infection, pain in the sitting position, and difficulty in taking care of the baby. This study aimed to investigate the effect of Camellia sinensis ointment on perineal pain and episiotomy wound healing in primiparous women.

**Methods:**

This triple-blinded randomized clinical trial was conducted on 60 primiparous women who were referred to the maternity ward of Al-Hadi hospital in Shoushtar and Ganjovian hospital in Dezful, Iran, from 2020 to 2021. Participants were randomly assigned into two groups of intervention (Camellia sinensis extract ointment) and control (placebo) with a follow-up of 14 days. REEDA scale (redness, edema, ecchymosis, discharge, and approximation) was used to measure wound healing and the Visual Analog Scale (VAS) was used to measure the pain intensity.

**Results:**

There was no significant difference between two groups before intervention in terms of sociodemographic characteristics, pain intensity, and episiotomy wound status. Scores of pain intensity and wound healing reduced on days 7, 10, and 14 post-intervention in the intervention group compared to placebo. There was a significant decrease between the groups of intervention and control in terms of the mean score of pain intensity (VAS scale) on day 10 (1.33 ± 0.71, 1.77 ± 0.93) and day 14 (0.73 ± 0.74, 1.13 ± 0.81) post-intervention (P < 0.05). Also, on day 14 post-intervention, there was a significant decrease between the groups of intervention and control in terms of the mean score of episiotomy wound healing (REEDA index) (0.53 ± 0.77, 1.77 ± 1.46) (P < 0.05). The GLM test was applied for repeated measures. REEDA index and VAS scale changed during different times (time-variable) (p < .001). But, the studied groups (group variable) and the studied groups (interaction effect of group * time) did not affect the changes in the REEDA index (p = .292, p = .306) and VAS scale (p = .47) during different times.

**Conclusion:**

Our study showed that Camellia sinensis extract ointment has a small effect on the healing process and pain reduction of episiotomy wounds. to confirm its effect, a study with a larger sample size should be conducted.

**Trial registration:**

This trial was registered in the Iranian Registry of Clinical Trials on 04/10/2019 with the IRCT ID: IRCT20190804044428N1. Participants were enrolled between 11 April 2020 and 20 January 2021. URL of registry: https://en.irct.ir/trial/41326.

## Introduction

Episiotomy is a surgical incision to the perineum made during childbirth to enlarge the vaginal opening for birth [1], facilitate the delivery of the fetal head, and reduce the pressure of the fetal head on the pelvic floor tissues [2]. Episiotomy is one of the most frequently performed procedures on women worldwide [3]. Although the use of episiotomy is decreasing in developed countries, it still remains high in developing countries. Since Asian women have shorter perineum lengths and stronger tissue, they are more prone to increase risk of tears. Therefore, episiotomy is still routinely performed in these countries [4, 5]. The prevalence of episiotomy varies significantly between countries [6], Its prevalence was found to be 8% in Netherlands, 20% in UK, 40.6% in Austria, 54% in North America [7], and 54% in Turkey [8]. In Iran, the prevalence of episiotomy was found to be 41.5% in Shahroud, 88.7% in Sari and 97.3% in Tehran [9–11].

Episiotomy can lead to complications such as bleeding, infection, inflammation, edema, wound opening, and pain [9, 12]. Perineal pain is the most common complication of episiotomy, and its prevalence was found to be 96.4% on the first day, 63% on the first day of labor, and 25% on the 40th day after delivery [9, 13]. The pain caused by the perineal wound can negatively impact the relationship between the mother and her baby and sometimes prevent the establishment of an emotional relationship between them. Delay in wound healing increases the risk of infection and poor anatomical outcomes, leading to dangerously infection complications, and even death. Despite being rare, dangerous infectious shock with an estimated mortality rate of 10–15% and fatal necrotizing fasciitis still occur due to episiotomy site infection [14, 15].

The acceleration of the healing of perineal wounds allows the mother to return to her daily activities earlier, establish an emotional connection with her baby, and improve her quality of life after childbirth. Since delay in episiotomy wound healing is associated with an increased risk of infection, prevention of perineal wound infection is a core component of routine maternity care [14].

There are pharmacological and non- pharmacological measures to reduce perineal pain and heal wounds such as maintaining perineal area clean and keeping the wound dry [15, 16]. Non-pharmacological treatments include cold therapy, laser therapy, electrical stimulation, acupuncture, and pelvic floor exercises. Pharmacological treatments involve acetaminophen, mefenamic acid, epidural analgesia, lidocaine gel, and diclofenac sodium suppositories [13].

The use of medicinal plants for the treatment of wounds has a long history in many countries, including Iran [17]. The most important herbal medicinal products in wound healing include olive, lavender, aloe vera, chamomile, marigold, and cinnamon [13]. Due to fewer side effects, high diversity of effective compounds, cost-effectiveness, the development of industries related to the cultivation of herbal medicinal products, and the World Health Organization’s suggestion, many use herbal medicinal products for wound healing [18].

Camellia sinensis is a great antioxidant and can stop cancer cells from growing, exert anti-aging, and anti-inflammatory effects and affect immune responses [19]. Camellia sinensis contains several polyphenolic components with antioxidant properties, but the predominant active components are phenolic acids and catechins [20, 21]. Epigallocatechin gallate (EGCG) has antibacterial and antiviral properties to accelerate wound healing. EGCG trigger multiplication, division, and activation of natural cell growth through cell division and anti-apoptotic effects [18]. A small amount of EGCG can increase the volume of collagen to heal skin wounds and multiply and differentiation of keratinocytes [20]. Episiotomy is a wound in the perineum that needs to go through healing phases like any other wound in the body. The first phase is the hemostatic and inflammatory phase, which is accompanied by pain [22].

Perineal pain can hinder women from performing household tasks and maternal duties, as well as exacerbate postpartum mood changes [23]. Relieving pain and healing the episiotomy wound using methods with minimal side effects and greater effectiveness and acceptability is highly important. However, very few studies have examined the effect of Camellia sinensis ointment on relieving pain and healing episiotomy wounds. A study by Asadi et al. (2013) demonstrated Camellia sinensis could decrease wound healing duration and lead to granulation tissue containing less inflammatory cells and more collagen in rats [19]. A study by Shahrahmani et al., [13] in Kerman (one of the central cities of Iran) showed camellia sinensis ointment healed episiotomy wounds.

Since cultural factors such as weather conditions and lifestyle can affect health and disease, this study aimed to investigate the effect of Camellia Sinensis ointment on relieving pain and healing episiotomy wounds in primiparous women in cities of Shoushtar and Dezful (southwest of Iran). Health is determined by various factors, including genetic inheritance, personal behaviors, access to quality health care, and the environment (such as weather quality, housing conditions). In addition, health is related to individual, social and cultural factors. Along with the determinants of health and disease, culture can determine the perspective of patients and health care providers towards health and disease. The cultural characteristics of any group may be directly or indirectly related to priorities, decisions, behaviors, or to the acceptance and adoption of health and health education programs and messages. For example, the traditional food practices of a cultural group can promote or prevent some diseases [24].

## Materials and methods

### Study protocol

This clinical trial was reported based on the CONSORT 2010 checklist [25]. The study was approved by the Ethics Committee of the Shoushtar Faculty of Medical Sciences, Shoushtar, Iran (Reference number: IR.SHOUSHTAR.REC.1398.004). This trial was registered in the Iranian Registry of Clinical Trials on 04/10/2019 with the IRCT ID: IRCT20190804044428N1. Participants were enrolled between 11 April 2020 and 20 January 2021. URL of registry: https://en.irct.ir/trial/41326

### Study design

This study was a randomized, triple-blind clinical trial with two parallel groups of intervention and placebo.

### Participants and setting

This study was conducted on 60 primiparous women who were referred to maternity ward of Al-Hadi hospital in Shoushtar and Ganjovian hospital in Dezful, Iran, from 2020 to 2021.

### Inclusion and exclusion criteria

Inclusion criteria included primiparous mother, the age range of 18–35 years, being literate, full-term pregnancy with a live singleton fetus and head presentation, infant weight of 2500-4000g, body mass index (BMI) between 29.9–19.8 kg/m2, and mediolateral episiotomy (3 to 5 cm).

Exclusion criteria included abnormal vaginal bleeding and transfer of the mother to the operating room, or sensitivity to it, initiation of sexual intercourse during 10 days after labor, infection of the episiotomy site and opening of the wound, extension of the length of episiotomy incision site and turning into a 3rd and 4th-degree tear, shoulder dystocia (which leads to the use of maneuvers other than McRobert’s), hospitalization of the baby in the intensive care unit (ICU), use of effective medications on wound healing by the mother (anticoagulants, antidepressants, antiepileptics, alcohol, glucocorticoids, immune system suppressants, antibiotics and chemotherapy), drugs and psychoactive drugs, having diseases interfere with wound healing (chronic systemic, cardiac, renal, pulmonary diseases, coagulation disorder, immune deficiency, connective tissue disorder, diabetes, severe anemia, mental illness, hemophilia, depression, malnutrition), having visible lesions in the perineum (genital warts, hemorrhoids), having persistent constipation (according to the patient), rupture of the amniotic sac longer than 18 hours, non-spontaneous expulsion of the placenta (manual expulsion), perineal hematoma, reoperation perineum after childbirth, absence of severe cystocele and rectocele, and having a history of reconstructive surgery of vagina and perineum.

### Sample size

The sample size was based on the research objective (comparison of pain according to the VAS index in two groups at the end of the study), and a previous study [26]. Considering d = 3.2, $s_{2}^{2}=4.9$, $s_{1}^{2}=3.4$, β = 0.2, α = 0.05, and using the average comparison formula and taking into account the attrition rate of 10%, the calculated sample size was 60 participants (30 in each group).

### Randomization and blinding

In order to control the confounding variables, samples were randomly assigned into two groups: intervention (Camellia sinensis extract ointment) and control (placebo) with a follow-up of 14 days.

In this study, participants were selected using a convenience sampling method. Cards were placed in two colors with code 1 and code 2 in sealed envelopes, and these envelopes were placed in a bag. Each eligible participant received a sealed envelope from the bag. The envelope contained a card indicating both their group assignment (simple randomization) and the type of tube to be used (ointment or placebo), determined by a coded label within (allocation concealment). The tubes, provided by the pharmacist, were visually identical with codes labeled as either ’Code 1’ or ’Code 2.’ The researcher, participants, and statistician were blinded to the codes and remained unaware of the corresponding type of drug. This ensured both randomization and allocation concealment. A total of 80 sealed envelopes were placed inside the bag (Taking into account the attrition rate, 40 cards were allocated for each code).

The ointment code was recorded in the patient evaluation form. Researchers, the study participants, and the statistical specialist who analyzed the data were blinded to the procedure. At the end of the study, blind codes were decoded and patients who were assigned to each group were identified. Consent was obtained from the participants after explaining the study objectives and procedures and their right to refuse not to participate in the study any time they want was also assured.

### Data collection tools

Data collection tools included demographic characteristics form (e.g. mother’s age, mother’s education status, spouse’s education status and Income level), obstetric characteristics form and labour characteristics form (e.g. gestational age, number of gravida, abortion, baby weight (g) and baby’s sex), daily form of medication use registration, medication side effect form, REEDA scale, and VAS Scale.

The REEDA scale was used to assess episiotomy wound healing. This scale was developed by Davidson in 1974 to assess episiotomy wound healing through the evaluation of five items of healing redness, edema, ecchymosis, discharge, and approximation of the wound edges [27]. This scale included five items and a score between 0 and 3 was assigned to each item. The total score on the scale ranged from 0 (maximum improvement) to 15 (minimum improvement). The validity and reliability of this scale were confirmed using the content validity method and Cronbach’s alpha, according to previous studies [26, 28]. After collecting the data using Cronbach’s alpha method, the reliability of REEDA scale was re-examined, and its reliability was confirmed (Cronbach’s Alpha = 0.81).

VAS scale was used to measure perineal pain intensity. The VAS uses a 10 cm line with endpoint descriptors such as ‘no pain’ marked at the left end of the line and ‘worst pain imaginable’ marked at the right end. A VAS score between 4–7 represents “mild pain”, a score between 4–7 represents “moderate pain” and a score between 8–10 represents “severe pain” [29]. The validity and reliability of this scale were confirmed in a previous study. The correlation and reliability of the scale was found to be 0.71–0.87, and r>0.8 and P<0.01, respectively [30]. The reliability of the VAS scale was re-examined and its reliability was confirmed (Cronbach’s alpha = 0.84).

#### Interventions and outcomes

In order to prepare the extract, Camellia sinensis was procured from the Tehran pharmaceutical market in November 2019. The plant’s identity was confirmed by the herbarium of School of Pharmacy, Shahid Beheshti University of Medical Sciences, Tehran, Iran (Herbarium number: 8253). Then, the plant was ground and made into a soft powder, and was extracted using the maceration method with 96° ethanol for 3 consecutive days. Afterward, the resulting extract with a concentration of 10% was mixed with an ointment base (cholesterol vaseline) and prepared in the form of 50-gram tubes. The placebo ointment was made by a pharmacist and contained a simple base ointment. Then the ointment and placebo ointment were coded (Code 1 and 2). The shape and appearance of the tubes were completely the same.

All research units were similar in terms of the type of episiotomy, method of repair, type of thread used, amount of anaesthetic before cutting and repair, and delivery factor. Deliveries and episiotomy repairs were performed by two midwives of the research group (one in Shushtar and one in Dezful). In cases that the duration of the second stage of labour or episiotomy repair time was more than usual (which lasted more than two hours without an epidural for nulliparous women) they were excluded from the study.

After delivery, the necessary trainings (e.g., information on the care of perineum and sutures, personal health, nutrition, amount of physical activity, how to apply the ointment, the time and place of next referrals, and the telephone number of the researcher) were given to the both groups alike in the form of educational packages by the researcher in person. The mothers in both groups were asked to wash their hands and perineum, dry the area, apply ointment enough to cover the entire length of the episiotomy wound, and use the cleansed sanitary pad after 5 min. They were required to apply the cream twice a day once in the morning and once in the night before going to bed for 14 consecutive days postpartum.

The mothers applied the first dose of the ointment in the first 24 hours postpartum (at least 2 hours after episiotomy repair) in the hospital under the surveillance of the researcher so that any reaction and sensitivity to the ointment were examined and recorded by the researcher. If participants had pain, the research units were allowed to use Mefenamic acid 250 mg capsules after determining pain level and using the VAS scale.

The mothers were requested to contact the researcher (if necessary) in the event of fever and shivering, sensitivity to the cream in the wound area, severe pain, swelling, burning sensation, itching, stiffness, dryness, and purulent discharge in the wound area. In case of such complications, they were asked to refer to the hospital and record them in the drug side effect reporting form.

To examine the appearance of an episiotomy wounds, the researcher contacted the mothers one day before the scheduled times for examination and reminded them of the place and time of the visits. Episiotomy wound healing rate was assessed in lithotomy position by the researcher using the REEDA scale and VAS scale on days 4, 7, and 10 postpartum using examination light. The oral temperature of mothers was measured during each visit and an interview and examination form was completed for each participant. All of them received routine care, including adherence to hygiene, wound care, and the physician’s prescribed medicines during the study period.

#### Statistics analysis

Data were analyzed using SPSS software (Version 23). Shapiro-Wilks test was used to examine the normality of data distribution. Due to the non-normal distribution of the data, Mann-Whitney, Chi-Square and Friedman tests were used to compare the studied groups. In addition, based on the general linear model (GLM), repeated measurements were used to define the factors. Based on the test of repeated measurements, we examined the effect of time, group and time-group interaction on REEDA and VAS scales. REEDA scale was used to assess wound healing and VAS was used to assess the pain intensity. A p-value less than 0.05 (P < 0.05) was considered statistically significant.

#### Ethics statement

All procedures performed on human samples were conducted in accordance with the relevant guidelines and regulations of the Helsinki Declaration. The study was approved by the Ethics Committee of the Shoushtar Faculty of Medical Sciences, Shoushtar, Iran (Reference number: IR.SHOUSHTAR.REC.1398.004). The informed written consent was obtained from all participants prior to data collection, and were informed about the study objectives and procedures. Moreover, the participants were ensured of anonymity and confidentiality of the data. The participants were permitted to withdraw from the study at any time. Participant information is kept in locked file cabinets.

## Results

**Fig 1:**
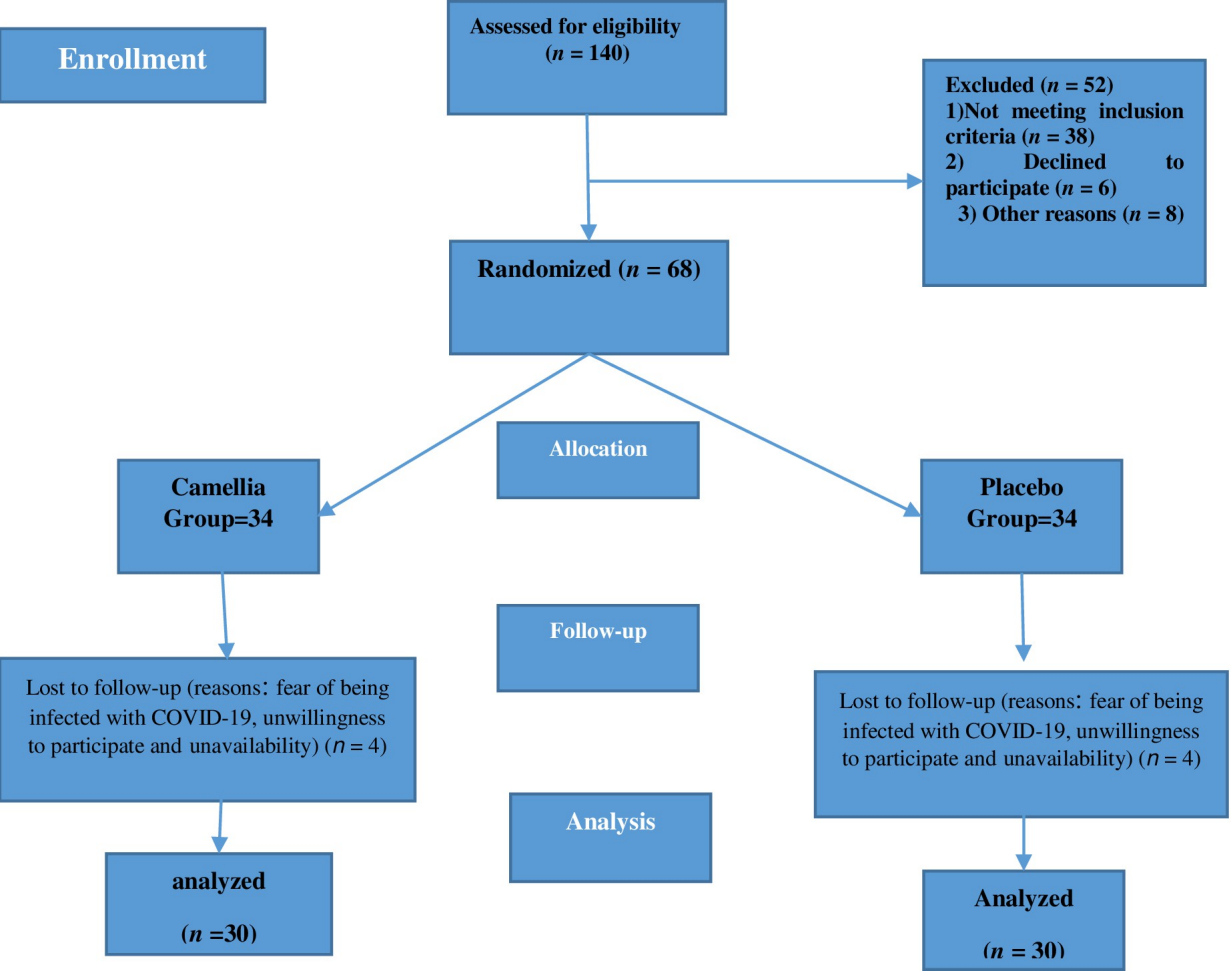
Flowchart of study participants.

A total of 68 primiparous women were randomly assigned into the Camellia sinensis and placebo groups. During the study, 8 participants were excluded due to lack of referral (fear of being infected with COVID-19) (n = 3), unwillingness to participate (n = 3) and unavailability (n = 2). Eventually, 60 women (30 in each group) were recruited (Fig 1).

According to Table 1, 57% and 43% of participants in the groups of intervention and control had high-school diplomas, respectively. Most of the participants in both groups had moderate-good income level. More than half of the women’s spouses (54.2%) in the control group had high-school diploma. Approximately 53% of women in the intervention group had male offspring. There was no a statistically significant difference between the two groups in terms of demographic characteristics (e.g. age, education status, employment status, household’s income) and fertility (e.g. gestational age, baby weight, number of pregnancies and abortions, and baby’s sex) characteristics (Table 1).

**Table 1:** Comparison of sociodemographic characteristics of participants in two groups of intervention and control.

Variable		Intervention group (n = 30) Mean ± SD/ frequency (%)	Control group (n = 30) Mean ± SD/ frequency (%)	P-Value
**Women’s age (year)**		28.87 ± 6.13	28.30 ± 4.57	0.97*
**Women’s education status (Diploma)**		12 (57.1)	9 (42.9)	0.65**
**Spouse’s education status (Diploma)**		11 (45.8)	13 (54.2)	0.43**
**Income level**	**Low**	2 (22.2)	7(77.8)	0.14**
	**Moderate**	4 (80.0)	1 (20.0)	
	**Good**	15 (57.7)	11(42.3)	
	**High**	9 (45.0)	11(55.0)	
**Number of gravida**		1.07 ± 0.25	1.03 ± 0.18	0.55*
**Number of abortion**		0.20 ± 0.48	0.25 ± 0.07	0.22*
**Gestational age (week)**		38.30 ± 1.26	38.57 ± 0.93	0.37*
**Baby weight (g)**		3311.67 ± 313.95	3252 ± 322.19	0.26*
**Baby’s sex (Boy)**		16 (53.3)	14 (46.7)	0.60**

*Mann-Whitney U

**Pearson Chi-Square

The results of this study showed that before the intervention, the mean scores of REEDA were not significantly different in the intervention and control groups (P  =  0.66). The mean score of episiotomy wound healing decreased on days 7 and 10 post-intervention in both intervention and control groups; however, the decrease was higher in the intervention group. There was no statistically significant difference in the mean score of REEDA on days 7 and 10 post-intervention between the two groups. Also, a statistically significant difference was found in the mean score of REEDA on day 14 post-intervention between the intervention and placebo groups (P < 0.05). The decrease was higher in the intervention group (Table 2).

**Table 2:** Comparison of the study groups in terms of the mean score of REEDA scale, on days 1,7,10 and 14 post-intervention.

Day	Intervention group (n = 30) Mean ± SD	Control group (n = 30) Mean ± SD	Mean Difference (Std. Error Difference)	95% Confidence Interval of the difference	P-Value Mann-whitney U
**Before intervention (the first day)**	4.17 ± 1.34	4.47 ± 1.43	-.30±.35	(-1.01,.41)	0.66
**Seven days after intervention**	2.97 ± 1.15	3 ± 1.50	-.03±.34	(-.72,.66)	0.62
**Ten days after intervention**	1.50 ± 1.16	1.70 ± 1.41	-.20±.33	(-.87,.47)	0.55
**Fourteen days after intervention**	0.53 ± 0.77	1.17 ± 1.46	-.63±.30	(-1.23, -.02)	0.04*
**The difference between the 1st and 14th day**	3.63±1.42	3.30±1.29	.33±.35	(-.36,1.03)	0.28
**P-value** **Friedman**	< .001	< .001	-	-	-

* Significance level: P < 0.05

As shown in Table 3, before the intervention, the mean scores of VAS scale were not significantly different in the intervention and control groups (P  =  0.36). There was no statistically significant difference in the mean score of VAS scale on day 7 post-intervention between the two groups. The mean score of pain intensity significantly decreased on days 10 and 14 post-intervention in both intervention and control groups. The decrease was higher in the intervention group.

**Table 3:** Comparison of the study groups in terms of the mean score of VAS scale, on days 1,7,10, and 14 post-intervention.

Day	Intervention group (n = 30) Mean ± SD	Control group (n = 30) Mean ± SD	Mean Difference (Std. Error Difference)	95% Confidence Interval of the difference	P-Value Mann-whitney U
**Before intervention (the first day)**	3.63 ± 0.66	3.73 ± 0.64	-.10±.16	(-.43,.23)	0.36
**Seven days after intervention**	2.47 ± 0.82	2.57 ± 0.85	-.10±.21	(-.53,.33)	0.50
**Ten days after intervention**	1.33 ± 0.71	1.77 ± 0.93	-.43±.21	(-.86, -.004)	0.03*
**Fourteen days after intervention**	0.73 ± 0.74	1.13 ± 0.81	-.40±.20	(-.80,.003)	0.04*
**The difference between the 1st and 14th day**	2.90±.80	2.60±.77	.30±.20	(-.10,.70)	0.15
**P-value Friedman**	< .001	< .001	-	-	-

* Significance level: P < 0.05

To compare the REEDA and VAS scales in the intervention and control groups, it is necessary to consider their results at different times. The GLM was applied for repeated measures. Box’s M test confirmed the homogeneity of the variance-covariance matrix (Box’s M = 16.71; P = 0.116) and (Box’s M = 4.67; P = 0.931). REEDA index changed during different times (time-variable) (p < .001). The studied groups (group variable) and the studied groups during different times (interaction effect of group * time) did not affect the changes in the REEDA index (p = .292, p = .306). The VAS scale changed during different times (time-variable) (p < .001). There was a difference between the two groups (group variable) (p < .001). But the studied groups (interaction effect of group * time) did not affect the changes of the VAS scale (p = .47) during different times (Table 4).

**Table 4:** Examination of the effect of treatment groups, different times and the effect of group* time, for VAS and REEDA indicators.

Variable	Effects	Sum of square	df	Mean square	F	p-value	Partial Eta squared
REEDA	Group	5.10	1	5.10	1.13	.29	0.01
	Time	416.54	1	416.54	434.61	< 0.001‎	0.88
	Group * time	1.021	1	1.021	1.06	.30	0.01
VAS	Group	4.004	1	4.004	2.47	.12	0.04
	Time	254.84	1	254.84	672.82	‎< 0.001‎	0.92
	Group * time	1.14	1	1.14	3.01	.08	0.04

## Discussion

To the best of our knowledge, this is the first study to examine the effect of Camellia sinensis ointment on healing and relieving wound pain in humans. This study was designed to investigate the effect of Camellia sinensis ointment on pain relief and episiotomy wound healing in primiparous women. The results of this study showed that the rate of wound healing on day 14 and pain relief on days 10 and 14 was higher in the intervention group than in the control group. REEDA’s index and VAS scale decreased over time, but this decrease was not statistically significant between the two groups during different times. Our results also showed the intervention group had a small effect on reducing the REEDA’s index and VAS scale compared to the control group (approximately 0.3), This finding is of importance clinically.

Our results were in line with previous studies that examined the effect of Camellia sinensis on wound healing. Karimi et al. investigated the effects of camellia sinensis ethanolic extract on histometric and histopathological healing process of burn wound in Rat. Their results showed that Camellia sinensis ointment could help wound healing process [31]. Shahrahmani et al. investigated the effect of Camellia sinensis ointment on perineal wound healing in primiparous women, and demonstrated Camellia sinensis ointment could improve episiotomy wound healing [32]. In our study, we observed improvement at certain times (wound healing on day 14 and pain relief on days 10 and 14). However, we observed a slight difference over time according to GLM, which was not statistically significant. The discrepancies in the results can be due to the use of different statistical tests (with more accuracy), differences in size, structure of the study population, and setting. Also, Kouhihabibidehkordi et al. investigated the effect of white tea (Camellia sinensis) extract on the skin wound healing process in rats. Their results showed that 5% white tea extract ointment could not help the skin wound healing process, which aligns with our results [33].

Polyphenols, catechins, and EGCG in Camellia sinensis cause the proliferation of fibroblasts and can affect their functional capacity and increase their ability to synthesize collagen fibers. Polyphenols cause induction, differentiation, and cell proliferation in epidermal keratinocytes, and also impede the secretion of interferon-gamma, and have anti-inflammatory, anti-aging, and wound-healing effects [18]. An Iranian study examined the effect of Camellia sinensis mucoadhesive paste on recurrent aphthous stomatitis treatment and showed the use of the mucoadhesive paste reduced the pain intensity and wound healing process, but it did not affect the size of lesions [28].

Among the studies conducted on relieving episiotomy pain using herbal medicines with properties similar to Camellia sinensis, which has flavonoid compounds, Biromvand et al. investigated the effect of gel containing the extract of anzerot plant on the severity of episiotomy pain in primiparous women. Their results showed that the pain intensity in the intervention group was lower on days 5–7 &10–12 compared to the placebo group [34], which is consistent with our results. We observed a decline in episiotomy wound pain on days 10 & 14. Baghal et al. investigated the effect of Plantago major cream on pain intensity and episiotomy wound healing on 107 nulliparous women. They showed, the intensity of pain and episiotomy wound healing in the intervention group was significantly different compared to the placebo group on days 3&7 [16], which was not consistent with our study. The difference can be due to the difference in other compounds in plantago major compared to Camellia sinensis and the different sample size.

The results of our study showed that Camellia sinensis ointment is well tolerated, and no side effects were reported by participants, which is in line with a previous study [32], and can confirm the safety of this plant in women.

### Strength and limitations

Our study has strengths and limitations that deserve to be mentioned. Its main strength lies in finding herbal medicinal to relieve the pain intensity and wound healing process, which has many psychological, economic, social, and health effects. Faster wound healing can reduce the risk of infection, allow the mother to return to her normal routine sooner, and improve her quality of life, allowing her to care for her baby and perform daily tasks more easily. It has been shown that rapid wound healing can prevent postpartum depression [31]. Another strength of the study is the use of a triple-blindness design and random assignment of samples to Camellia sinensis ointment and placebo groups, which reduced bias and controlled confounding factors.

The main limitation of the study was that due to the spread of the COVID-19 outbreak, mothers postponed their treatment process or did not refer to the hospitals (because of their fear of catching the virus and contagion for their babies) at the appointed times for examination of the episiotomy wound. To overcome this limitation, the participants were given a card with the date, time, and place of the next visit. Also, the researcher called the participants one day before the date of the visit and reminded their visit. In each visit, the participants were taught the necessary health tips to prevent COVID-19 infection. To prevent the participants from getting the infection, the wound examination was done in a private room in the ward without delay. Another limitation was the effect of the midwife or physician who performed the delivery on the episiotomy repair and wound healing. To overcome this limitation, two midwives of the research team performed all deliveries and episiotomy repairs. The stages of labour and the difference in their duration in different individuals (which can be considered a limitation) can impact the wound’s healing process and the study’s results. In order to control this factor (stages of labour as a confounder), in cases where the delivery was prolonged or the second stage lasted longer than the standard time (the duration of the second stage of labour or episiotomy repair time was more than usual which lasted more than two hours without an epidural for nulliparous women), individuals were excluded from the study.

The small sample size was another limitation of our study which was due to the spread of Covid-19 pandemic that restricted the access to the participants. Considering the safety of the drug and the absence of side effects in people using the drug, it is suggested to conduct a study with a larger sample size.

## Conclusion

Our study showed that Camellia sinensis ointment had a small effect on the wound healing and episiotomy pain relief. Considering that participants reported no side effects, and it did not interfere with breastfeeding, baby care, and daily activities. Therefore, it is recommended that further studies be conducted with a larger sample size and among individuals with moderate and severe pain and wounds to more accurately investigate the effect of camellia sinensis on pain relief and wound healing.

## Supporting information

S1 Checklist Reporting checklist for randomised trial. (DOCX)

S2 Checklist CONSORT 2010 checklist of information to include when reporting a randomised trial*. (DOC)

S1 File (DOC)

## Abbreviations

REEDA: Redness, Edema, Ecchymosis, Discharge, Approximation of the wound edges
VAS: Visual Analogue Scale
EGCG: Epigallocatechin gallate
BMI: Body Mass Index
CONSORT: Consolidated Standards of Reporting Trial
ICU: Intensive Care Unit
