# Supplementary material for: The effect of Camellia sinensis ointment on perineal pain and episiotomy wound healing in primiparous women: A triple-blind randomized clinical trial
Source: PLoS One. 2024 Aug 1;19(8):e0305048. doi: 10.1371/journal.pone.0305048 (PMC11293732; doi:10.1371/journal.pone.0305048)
Supplement: S1 File — (DOC) [file pone.0305048.s003.doc]

**The effect of Camellia sinensis (green tea) ointment** **on perineal pain and episiotomy wound healing in primiparous women: a triple-blind randomized clinical trial**

**Abstract**

**Background:**

Medicinal plants are widely used to treat wounds, among these plants is camellia sinensis. Considering that there is not enough evidence on the effectiveness of this plant on the healing of episiotomy wounds, this clinical trial aims to investigate the effect of Camellia sinensis (green tea) communis on perineal pain and episiotomy wound healing in primiparous women.

**Methods:** The design of the planned study is a triple-blinded randomized clinical trial. A total of 60 primiparous women will be selected and included in the study, 30 for each group. Participants were randomly assigned into two groups of intervention (receiving camellia sinensis extract ointment) and control (placebo) with a follow-up of 14 days.

Participants will be randomly assigned into intervention group (receiving camellia sinensis extract ointment) and control (placebo) with a follow-up of 14 days. The allocation of the participants in the intervention or control group is based on their randomly selection of cards with codes 1 and 2 will be placed in a bag. Participants, researcher, examiner (midwife) and analyzer are unaware of the type of interventions. REEDA scale will be used to measure wound healing and the Visual Analog Scale (VAS) will be applied to measure the pain intensity. The collected data will be analyzed by SPSS-23 software at a 95% confidential level.

**Conclusions:** The results of this study can be used to develop evidence to provide an effective intervention and a complementary treatment for pain relief and episiotomy wound healing in primiparous women.

**Keywords:** wound healing, pain intensity, episiotomy, green tea

**Introduction**

Episiotomy is a surgical incision made in the perineum during childbirth to enlarge the vaginal opening (1), facilitate the expulsion of the fetus, and reduce the pressure of the fetal head on the pelvic floor tissues (2). Episiotomy is one of the most common obstetric procedures (3). Although the use of episiotomy is decreasing in developed countries, it still remains high in developing countries. Since Asian women have shorter perineum lengths and stronger tissue, are more prone to increase risk of tears. Therefore, episiotomy is still routinely performed in these countries (4, 5). The prevalence of episiotomy varies significantly between countries (6), Its prevalence was found to be 8% in Netherlands, 20% in UK, 40.6% in Austria, 54% in North America (7), and 54% in Turkey (8). In Iran, the prevalence of episiotomy was found to be 41.5% in Shahroud, 88.7% in Sari and 97.3% in Tehran (9-11).

Episiotomy can create complications such as bleeding, infection, inflammation, edema, wound opening, and pain (9, 12). Perineal pain is the most common complication of episiotomy, and its prevalence was found to be 96.4% on the first day, 63% on the first day of labor, and 25% on the 40th day after delivery (9, 13). The pain caused by the perineal wound can negatively impact the relationship between the mother and her baby and sometimes prevents the establishment of an emotional relationship between them. Delay in wound healing increases the risk of infection and poor anatomical outcomes, leading to dangerously infection complications, and even death. Despite being rare, dangerous infectious shock with an estimated mortality rate of 10-15% and fatal necrotizing fasciitis still occur due to episiotomy site infection (14, 15).

Accelerating the healing of perineal wounds allows the mother to return to her daily activities earlier, establish an emotional connection with her baby, and improve her quality of life after childbirth. In contrast, delay in episiotomy wound healing is associated with an increased risk of infection, therefore, preventing perineal wound infection is a core component of routine maternity care (14).

***1-1- Evidence-based practice in*** ***episiotomy wound care***

In recent decades, many pieces of pharmacological and non- pharmacological evidence for reduce perineal pain and wound healing were developed; these practices include a wide range of interventions such as maintaining perineal area clean, keeping the wound dry, and using different treatments (15, 16). Non-pharmacological treatments include cold therapy, laser therapy, electrical stimulation, acupuncture, and pelvic floor exercises. Pharmacological treatments involve acetaminophen, mefenamic acid, epidural analgesia, lidocaine gel, and diclofenac sodium suppositories (13). The use of medicinal plants for the treatment of wounds has a long history in many countries, including Iran (17). The most important herbal medicinal products in the wound healing include olive, lavender, aloe vera, chamomile, marigold, and cinnamon (13). Due to fewer side effects, high diversity of effective compounds, cost-effectiveness, the development of industries related to the cultivation of herbal medicinal products, and the World Health Organization's suggestion, many use herbal medicinal products for wound healing (18).

Green tea has been studied for its effect as a potent antioxidant and cancer-prevention agent and has been shown can exert anti-aging, and anti-inflammatory effects and inducing changes in immune responses (19). Green tea extract contains several polyphenolic components with antioxidant properties, but the predominant active components are phenolic acids and catechins (15, 16). Epigallocatechin gallate (EGCG) has antibacterial and antiviral properties to accelerate wound healing. EGCG trigger multiplication, division, and activation of natural cell growth through cell division and anti-apoptotic effects (18). A small amount of EGCG can increase the volume of collagen to heal skin wounds and multiply and differentiation of keratinocytes (15).

***1.2. Critical Appraisal for Context***

In Iran, episiotomy is performed for almost all primiparous vaginal delivery (9-11). Perineal pain makes women to be unable to perform housework and maternal duties and aggravates mood changes after childbirth (20). Chemical drugs are often used to heal episiotomy wounds and pain. Considering the extent of herbal medicines and the background of medicinal plants and the acceptability of their use among Iranians, it is possible to replace chemical drugs with herbal medicinal plants with less side effects. Relieving pain and healing the episiotomy wound using methods with minimal side effects and greater effectiveness and acceptability is highly important. however, very few studies have examined the effect of camellia sinensis ointment on relieving pain and healing episiotomy wounds.

The research team decided to investigate the effect of Camellia sinensis (green tea) ointment extract on relieving pain and healing episiotomy wounds in primiparous women. However, evidence to support their clinical efficacy in relieving pain and healing episiotomy wounds is limited.

**2.Materials and methods**

**2.1. *Aim***

The aim of this study is to investigate the effect of Camellia sinensis (green tea) ointment on perineal pain and episiotomy wound healing in primiparous women.

**2.2. Study design**

This is a randomized triple-blinded clinical trial.  Each eligible patient will be allocated to one of the two groups: ”intervention group” and “placebo group” , based on the random selection of coded cards (code 1 and code 2) by patients from inside the bag.

- Group A: Patients received camellia sinensis extract ointment

- Group B: Patients received placebo

The study will be approved by the Shoushtar Faculty of Medical Sciences after confirming the proposal by the Ethics Committee at Shoushtar Faculty of Medical Sciences and registering in the Iranian Registry of Clinical Trials.

We will conduct the study according to the ethical principles of the Helsinki Declaration of 2013 and follow the CONSORT 2010 checklist (21). The study design is shown in Figure 1.

Enrollment

**Assessment of eligibility (n=…)**

Excluded (n= …)

- Not meeting inclusion criteria (n=…)
- Declined to participate (n=…)

## Permuted block randomized allocation (n=60)

**Group1** (n=30)

received camellia sinensis extract ointment

**Group2** (n=30)

received placebo

Analysis

Analyzed (n=30)

Analyzed (n=30)

Patients completed interventions 1 **(n=…)**

- Leaving follow-up (= 10%)

Patients completed interventions 3**(n=…)**

- Leaving follow-up (= 10%)

Analysis

**Analyzed (n ≥ 30 patients)**

**Analyzed (n ≥ 30 patients)**

**Figure 1. CONSORT flow diagram**

***2.3. Sample size calculation***

The sample size is calculating according to a previous study (22) with the following formula:

β = 0.5,

α=0.8


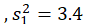


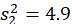


d=3.2


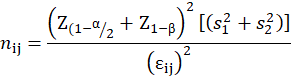


With an 80% power, and the attrition rate of 10%, the calculated sample size was 60, including 35 participants in each group.

***2.4. Participants and recruitment***

Patients in the two university-affiliated hospitals located in Shoushtar (Al-Hadi) and Dezful (Ganjovian), Iran, will be recruited:

***2.4.1. Inclusion criteria***

- Being primiparous

-Aged 18-35 years

-Being literate

-Full-term pregnancy with a live singleton fetus and head presentation

- Baby weight between 2500-4000 grams

- Body Mass Index (BMI) between 29.9 - 19.8 kg/m^2^

-Non-use of effective medications on wound healing by the mother (anticoagulants, antidepressants, antiepileptics, alcohol, glucocorticoids, immune system suppressants, antibiotics and chemotherapy), drugs and psychoactive drugs

- Not having diseases interfere with wound healing (chronic systemic, cardiac, renal, pulmonary diseases, coagulation disorder, immune deficiency, connective tissue disorder, diabetes, severe anemia, mental illness, hemophilia, depression, malnutrition)

- Not having visible lesions in the perineum (genital warts, hemorrhoids)

- Not having persistent constipation (according to the patient)

- No rupture of the amniotic sac longer than 18 hours

- Normal and spontaneous placental abruption

- No perineal hematoma

-No reoperation Perineum after childbirth, absence of severe cystocele and rectocele

- No history of reconstructive surgery of vagina and perineum

- Mediolateral episiotomy 3 to 5

***2.4.2. Exclusion criteria***

- Abnormal vaginal bleeding and transfer of the mother to the operating room

- Not using the ointment regularly and according to the instructions (more than two nights) or sensitivity to it

- Initiation of sexual intercourse during 10 days after labor

- Infection of the episiotomy site and opening of the wound

-Extension of the length of the incision site and turning into a 3rd and 4th-degree tear

-Shoulder dystocia (which leads to the use of maneuvers other than McRobert's)

-Hospitalization of the baby in the intensive care unit (ICU)

- Unwillingness to continue participating in the study.

***2.5. Implementation of the Interventions***

All patients will be received routine care including adherence to hygiene, wound care, and the use of medicines prescribed by the physician during the study period. In addition to this usual care, prior to applying the planned interventions, the researcher will present educational booklets (e.g., information on the care of perineum and sutures, personal health, nutrition, amount of physical activity, how to apply the ointment, the time and place of next referrals, and the telephone number of the researcher) as face-to-face and educational packages to all groups. The mothers will be asked to wash their hands and perineum, dry the area, apply ointment enough to cover the entire length of the episiotomy wound, and use the clean sanitary pad after 5 min. The mothers will apply the first dose of the ointment in the first 24 h postpartum (at least 2 h after episiotomy repair) in the hospital under the surveillance of the researcher so that any reaction and sensitivity to the ointment will be examined and recorded by the researcher. The mothers will be asked to contact the researcher (if necessary) in the event of fever and shivering, sensitivity to the cream or in the wound area, severe pain, swelling, burning sensation, itching, stiffness, dryness, and purulent discharge in the wound area. In case of the occurrence of such complications, they will be asked to refer to the hospital and record the complications in the complication registration form. The interventions are as below:

- For the intervention group, Camellia sinensis (green tea) ointment will be applied twice a day once in the morning and once in the night before going to bed for 14 consecutive days postpartum.
- For the control group placebo ointment will be applied similar to the intervention group.

***2.6. Outcome***

Assessments will be conducted using the statistical software SPSS version 23, setting the p-values at < 0.05. Data collection tools are included demographic characteristics form (e.g. age, education status, household’s income, etc.), daily form of medication use registration, medication side effect form, wound healing REEDA scale, and Visual Analogue Scale (VAS).

The wound healing REEDA scale was developed by Davidson in 1974 to measure episiotomy wound healing through the evaluation of redness, edema, ecchymosis, discharge, and approximation of the wound edges (23). This scale included five items and a score between 0 and 3 was assigned to each item. The total score on the scale ranged from 0 (maximum improvement) to 15 (minimum improvement). The validity and reliability of this scale were confirmed using the content validity method and Cronbach’s alpha, according to previous studies (22, 24, 25).

VAS is used to measure perineal pain intensity. The VAS uses a 10 cm line with endpoint descriptors such as ‘no pain’ marked at the left end of the line and ‘worst pain imaginable’ marked at the right end. A VAS score between 4-7 represents “mild pain”, a score between 4-7 represents “moderate pain” and a score between 8-10 represents “severe pain” (26). The validity and reliability of this scale were confirmed in a previous study. The correlation and reliability of the scale was found to be 0.71-0.87, and r>0.8 and P<0.01, respectively (27).

***2.6.1 Primary outcome***

To determine the appearance of an episiotomy wound, the researcher will contact the patients one day before the scheduled times for examination and reminded them of the place and time of the visit. Episiotomy wound healing rate will be assessed in lithotomy position by the researcher using the REEDA wound measurement scale and VAS on days 7,10,14 postpartum using examination light. The oral temperature of mothers will be measured during each visit and an interview and examination form will be completed for each participant.

***2.7. Data Analysis***

The results are analyzed using chi-square, t-test, Mann-Whitney, ANOVA, Pearson's correlation coefficient and multiple linear regression tests.

**3. Discussion**

This study investigate the effect of green tea extract on relieving pain and healing episiotomy wounds in primiparous women.

There are many pieces of evidence which show green tea extract could decrease wound healing duration considerably, and lead to granulation tissue containing less inflammatory cells and more collagen in rats (19, 28, 29). Polyphenols, catechins, and EGCG in green tea cause the proliferation of fibroblasts and affect the functional capacity of fibroblasts and increase the ability to synthesize collagen fibers. Polyphenols cause induction, differentiation, and cell proliferation in epidermal keratinocytes, and also impede the secretion of interferon-gamma, and have anti-inflammatory, anti-aging, and wound-healing effects (18).

An Iranian study examined the effect of green tea mucoadhesive paste on recurrent aphthous stomatitis treatment and showed the use of the mucoadhesive paste reduced the pain intensity and healing wound process, but it had no effect on reducing the size of lesions (25). There is a large space to generate evidence from high-quality research to employ evidence-based practice. This randomized triple-blinded clinical trial can work in this task.

This is randomized triple blinded clinical trial study to investigate the effect of traditional Iranian herb, green tea, on episiotomy wound healing, which can be used as a complementary treatment for pain relief and episiotomy wound healing in primiparous women.

The main strength of the study is the use of herbal medicinal to relieve the pain intensity and promote the wound healing process, which has many psychological, economic, social, and health effects. Faster wound healing can reduce the risk of infection, the mother returns to her normal routine sooner, and improve her quality of life, allowing her to care for her baby and perform daily tasks more easily. In several studies, the relationship between perineal wound inflammation and postpartum depression symptoms has been established, indicating that rapid wound healing can prevent postpartum depression (30). Another strength of the study is the use of a triple-blindness design and random assignment of samples to green tea extract ointment and placebo groups, which reduced bias and controlled confounding factors. The study limitation would be due to the probability of the attrition rates which been taken into account in the sample size calculation.

**Conclusion**

The results of the study seek to find herbal treatments without side effects or with fewer side effects than chemical drugs to heal episiotomy wounds, which can be replaced by chemical drugs.

**References**
